# Leapfrogging *Sycamore*: harnessing 1432 GPUs for 7× faster quantum random circuit sampling

**DOI:** 10.1093/nsr/nwae317

**Published:** 2024-09-12

**Authors:** Xian-He Zhao, Han-Sen Zhong, Feng Pan, Zi-Han Chen, Rong Fu, Zhongling Su, Xiaotong Xie, Chaoxing Zhao, Pan Zhang, Wanli Ouyang, Chao-Yang Lu, Jian-Wei Pan, Ming-Cheng Chen

**Affiliations:** Hefei National Research Center for Physical Sciences at the Microscale and School of Physical Sciences, University of Science and Technology of China, Hefei 230026, China; Shanghai Research Center for Quantum Science and CAS Center for Excellence in Quantum Information and Quantum Physics, University of Science and Technology of China, Shanghai 201315, China; Hefei National Laboratory, University of Science and Technology of China, Hefei 230088, China; Shanghai Artificial Intelligence Laboratory, Shanghai 200232, China; Shanghai Artificial Intelligence Laboratory, Shanghai 200232, China; Shanghai Research Center for Quantum Science and CAS Center for Excellence in Quantum Information and Quantum Physics, University of Science and Technology of China, Shanghai 201315, China; Hefei National Research Center for Physical Sciences at the Microscale and School of Physical Sciences, University of Science and Technology of China, Hefei 230026, China; Shanghai Research Center for Quantum Science and CAS Center for Excellence in Quantum Information and Quantum Physics, University of Science and Technology of China, Shanghai 201315, China; Hefei National Laboratory, University of Science and Technology of China, Hefei 230088, China; Shanghai Artificial Intelligence Laboratory, Shanghai 200232, China; Shanghai Artificial Intelligence Laboratory, Shanghai 200232, China; Shanghai Artificial Intelligence Laboratory, Shanghai 200232, China; Shanghai Artificial Intelligence Laboratory, Shanghai 200232, China; CAS Key Laboratory for Theoretical Physics, Institute of Theoretical Physics, Chinese Academy of Sciences, Beijing 100190, China; Shanghai Artificial Intelligence Laboratory, Shanghai 200232, China; Hefei National Research Center for Physical Sciences at the Microscale and School of Physical Sciences, University of Science and Technology of China, Hefei 230026, China; Shanghai Research Center for Quantum Science and CAS Center for Excellence in Quantum Information and Quantum Physics, University of Science and Technology of China, Shanghai 201315, China; Hefei National Laboratory, University of Science and Technology of China, Hefei 230088, China; Hefei National Research Center for Physical Sciences at the Microscale and School of Physical Sciences, University of Science and Technology of China, Hefei 230026, China; Shanghai Research Center for Quantum Science and CAS Center for Excellence in Quantum Information and Quantum Physics, University of Science and Technology of China, Shanghai 201315, China; Hefei National Laboratory, University of Science and Technology of China, Hefei 230088, China; Hefei National Research Center for Physical Sciences at the Microscale and School of Physical Sciences, University of Science and Technology of China, Hefei 230026, China; Shanghai Research Center for Quantum Science and CAS Center for Excellence in Quantum Information and Quantum Physics, University of Science and Technology of China, Shanghai 201315, China; Hefei National Laboratory, University of Science and Technology of China, Hefei 230088, China

**Keywords:** quantum advantage, quantum random circuit sampling, tensor network, parallel computing

## Abstract

Random quantum circuit sampling serves as a benchmark to demonstrate quantum computational advantage. Recent progress in classical algorithms, especially those based on tensor network methods, has significantly reduced the classical simulation time and challenged the claim of first-generation quantum advantage experiments. However, in terms of generating uncorrelated samples, time to solution and energy consumption, previous classical simulation experiments still underperform the *Sycamore* processor. Here we report an energy-efficient classical simulation algorithm, using 1432 GPUs to simulate quantum random circuit sampling that generates uncorrelated samples with a higher linear cross-entropy score and is 7$\times$ faster than the *Sycamore* 53-qubit experiment. We propose a post-processing algorithm to reduce the overall complexity, and integrate state-of-the-art high-performance general-purpose GPUs to achieve two orders of lower energy consumption compared to previous works. Our work provides the first unambiguous experimental evidence to refute *Sycamore*’s claim of quantum advantage, and redefines the boundary of quantum computational advantage using random circuit sampling.

## Teaser

This work presents an energy-efficient classical simulation algorithm using GPUs that outperforms Google's Sycamore processor in speed and energy consumption, challenging the claim of quantum computational advantage.

## INTRODUCTION

Quantum computers represent a new paradigm of computing, and in theory promise to solve certain problems much faster than classical computers [1,2]. A major milestone is to discover quantum algorithms for noisy intermediate-scale quantum computers and demonstrate the long-anticipated quantum computational advantage (QCA) [3]. Such algorithms include boson sampling and its variant [4–6], random circuit sampling (RCS) [7] and instantaneous quantum polynomial sampling [8]. Using superconducting circuits [9–12] and photons [13–16], increasingly sophisticated experiments have provided strong evidence of QCA. For example, in Google’s 2019 groundbreaking experiment, the *Sycamore* processor obtained one (three) million uncorrelated samples in 200 (600) s, with a linear cross-entropy (XEB) of 0.2% [9]. It was then estimated that simulating the same process on the *Summit* supercomputer would take 10 000 years [9]. XEB estimates circuit fidelity by comparing the sample distribution from experiments with theoretical predictions. We elaborate upon XEB, and the post-processing step to increase it, in the subsequent Methods section.

**Figure 1.**
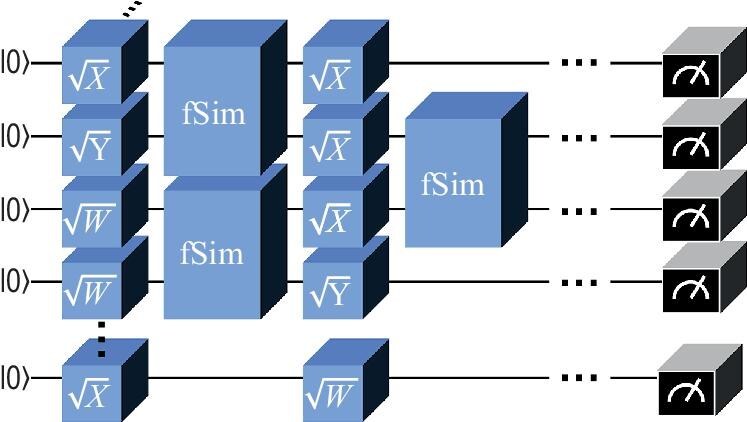
Schematic diagram of a quantum random circuit. The single-qubit gates are randomly selected from $\lbrace \sqrt{X}, \sqrt{Y}, \sqrt{W}\rbrace$. The random selection of single-bit gates creates a Haar-random ensemble to ensure average-case hardness [25], while the fSim gate facilitates entanglement between qubits and cannot be efficiently decomposed [18].

Similar to the Bell experiments, the QCA is not a single-shot achievement, but expects continued competition between improved classical simulation algorithms and upgraded quantum hardware. During this process, the QCA milestone can be progressively better established. For the random circuit sampling, shown in Fig. 1, emerging classical algorithms [17–22] based on tensor networks have significantly reduced the time to solution and mitigated the exponential growth of memory demands, and have challenged the claim of first-generation quantum advantage. These algorithms leverage the low-XEB value (0.002) of the *Sycamore* experiment to employ low-fidelity simulations, emerging as an effective strategy for reducing complexity.

**Figure 2.**
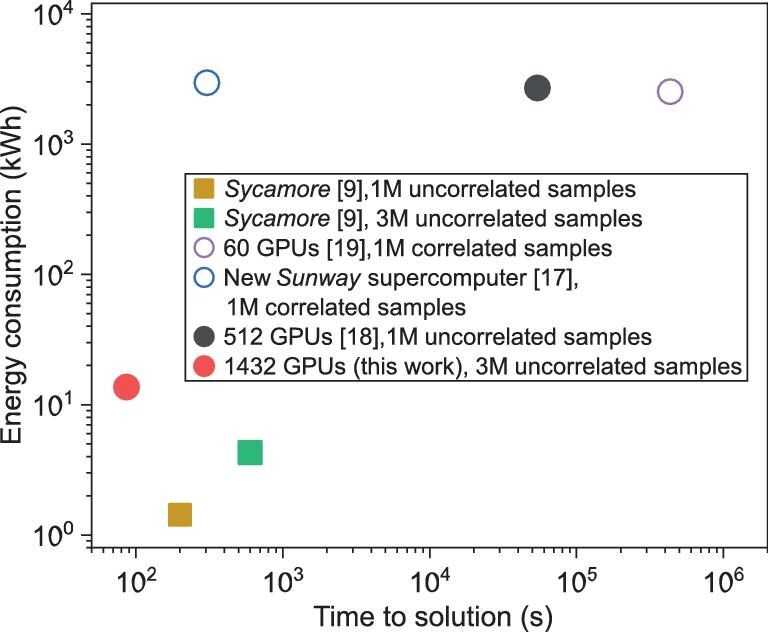
Performance of implementations of sampling the *Sycamore* circuit. The horizontal axis denotes the time to solution, and the vertical axis denotes the energy consumption in the quantum experiment or classical simulations. Circles and squares correspond to classical simulations and quantum experiments, respectively. The open circle indicates a correlated sampling loophole in the corresponding classical simulation.

Among the previous attempts, summarized in Fig. 2, Liu *et al.* [17] used the *Sunway* supercomputer to perform the classical simulation, and obtained one million samples in 304 s—still slower than *Sycamore*. More importantly, their obtained samples were *correlated*, which was not equivalent to a true quantum experiment. Gao *et al.* [20] completed the simulation in 0.6 s, by simplifying the circuit into two independent parts, which, however, resulted in a low-XEB value of $1.85\times 10^{-4}$, failing to meet the benchmark set by *Sycamore*. Pan *et al.* [18] employed 512 GPUs to successfully obtain one million *uncorrelated* samples [23] with an XEB value of 0.0037, but at a time cost of 15 h. We also comprehensively compare various classical simulation algorithms alongside the *Sycamore* experiment; see Table 1.

**Table 1.** Comparison of the time to solution, energy consumption, number of samples, sample correlation and the XEB fidelity across different platforms and algorithms for quantum random circuit sampling tasks with 53 qubits and 20 cycles.

	Time to solution (s)	Energy consumption (kWh)	Number of samples	Correlation of samples	XEB (%)
*Sycamore* [9]	600	4.3	$3\times 10^{6}$	Uncorrelated	0.2
New *Sunway* supercomputer [17]	304	2955	$1\times 10^{6}$	Correlated	–
60 GPUs [19]	$4.32\times 10^{5}$	2520	$1\times 10^{6}$	Correlated	1
512 GPUs [18]	$4.68\times 10^5$	2688	$1\times 10^{6}$	Uncorrelated	0.37
**1432 GPUs (our work)**	86.4	13.7	$3\times 10^{6}$	Uncorrelated	0.2

In addition to the time to solution, the XEB and the sample correlation, another factor worth mentioning is energy consumption [24]. The energy consumption of previous work [17–22] is approximately three orders of magnitude higher than the quantum processor. For practical reasons, reducing the power overhead is also of great interest.

## SUMMARY OF RESULTS

In this work, we propose a new algorithm that obtains samples with XEB values comparable to that of *Sycamore* and achieves better performance in terms of the running time and energy cost, thereby experimentally refuting Google’s quantum advantage claim [9] in both the solution time and the energy cost. Compared to previous high-XEB uncorrelated sampling works, we reduce the energy consumption by two orders of magnitude. Our algorithm uses partial network contraction to achieve a low-complexity approximation [26]. According to the insight of the Porter-Thomas distribution and a flaw in the XEB measure, we develop a post-processing algorithm to increase the XEB values. We also note that some works mentioned a similar method [19,20,27]. We find that the computational complexity of the optimal contraction scheme is approximately inversely proportional to the storage space, and develop an advanced 8-GPU parallel tensor contraction algorithm that increases the accessible storage space to $8 \times 80\, \mathrm{GB} =640 \, \mathrm{GB}$ to reduce the computational complexity without significantly increasing the communication time. Finally, using 1432 NVIDIA A100 GPUs, we obtain three million uncorrelated samples with XEB values of $2\times 10^{-3}$ in 86.4 s, consuming only 13.7 kWh of electricity in total. In contrast, *Sycamore* performs the same task and obtains three (one) million samples in 600 (200) s, consuming 4.3 kWh of electricity for cooling water [9].

## METHODS

### Composition of our algorithm

Our algorithm works in the following way. First, we calculate the approximate output probability $p(s)$ of $k$ bitstring groups by contracting a fraction $f$ of the tensor network, where each group includes $2^l$ correlative bitstrings. These preprocessed samples are generated by iterating over all $l$-bit strings for $l$ qubits, which are called open qubits, and sampling uniformly over the rest of qubits $k$ times. We obtain a sample sequence $\tilde{\mathcal {S}} = \lbrace s_{L}^{i}\!\mid\! L\in \lbrace 1,\dots ,k\rbrace ,i\in \lbrace 1,\dots ,2^l\rbrace \rbrace$. Then, based on the top-$k$ method, we obtain the desired sequence of samples $\mathcal {S}$ by selecting the elements in $\tilde{\mathcal {S}}$ with the top-$k$ largest ${p}(s)$ values. Finally, we obtain $k$ uncorrelated bitstrings, whose XEB is $f \ln (|\tilde{\mathcal {S}}|/k)$ (see Equation (4) below). We note that there are some works that also use a similar method to emulate *Sycamore* [19,20,27]. In our work, we give a more systematic proof both theoretically and numerically.

The tensor network is divided into many subnetworks via the slicing method [28–31], which is to fix the indices over certain edges for each subnetwork such that each subnetwork can be contracted independently and the space complexity for each contraction is reduced while increasing the total time complexity. We only contract a fraction of the subnetworks. The approximate simulation is similar to the idea in previous work [18]. This method significantly reduces the computational complexity at the expense of a decrease in fidelity.

Notably, as is discussed in detail later, the top-$k$ method amplifies the XEB value by distilling from $\tilde{\mathcal {S}}$ and, as a result, significantly alleviates the requirement on the fidelity of the classical simulation.

### Post-processing method and XEB amplification

In the quantum RCS problem, the fidelity of a sequence of samples $\mathcal {S}$ is estimated by the XEB [7], and the definition of XEB ($ \mathcal {F}_{{\rm XEB}}$) is


(1) \begin{eqnarray*}
\mathcal {F}_{{\rm XEB}}(\mathcal {S}) := \frac{N}{|\mathcal {S}|}\sum _{s\in \mathcal {S}}q(s) - 1,
\end{eqnarray*}

where $N=2^n$ is the number of all possible bitstring outcomes, with $n$ the number of qubits, and $q(s)$ is the ideal output probability for bitstring $s$. The ideal output probability $q$ is predicted to be Porter-Thomas (PT) probability distribution [7] given by $P(Nq) = e^{-Nq}$. If a large enough number of samples is obtained according to the ideal output probability then their XEB value should approach 1 [9]. In our algorithm, in an ideal setting where we calculate the probability distribution for each string in $\tilde{\mathcal {S}}$ accurately, $\mathcal {S}$ is obtained as the top-$k$ strings in $\tilde{\mathcal {S}}$ with the largest output probability. Using the fact that the distribution of the output probabilities approaches the PT distribution for deep circuits, we prove in the Appendix that


(2) \begin{eqnarray*}
\left\langle \mathcal {F}_{\text{XEB},\text{top-}k}^{{\rm ideal}}(\mathcal {S})\right\rangle = \ln (|\tilde{\mathcal {S}}|/|\mathcal {S}|)= \ln (|\tilde{\mathcal {S}}|/k).
\end{eqnarray*}

Hence, in the ideal case, the top-$k$ method amplifies the XEB value by about $\ln (|\tilde{\mathcal {S}}|/k)$. Moreover, for noisy quantum random circuits, the XEB value is argued to be a good approximation to the circuit fidelity [9]. As for classical simulations, since each subnetwork contributes equally to the final fidelity [32], we can achieve fidelity $F=f$ by summing over a fraction $f$ of all subnetworks. In this way, we expect that an approximate simulation would obtain samples with an XEB value of


(3) \begin{eqnarray*}
\left\langle \mathcal {F}_{\rm XEB}^{{\rm approx}}(\mathcal {S})\right\rangle = F= f,
\end{eqnarray*}

via Markov chain Monte Carlo (MCMC) sampling [18]. Based on Equation (2), we also expect that our algorithm, which combines approximate simulation with the top-$k$ method, can achieve an XEB value of


(4) \begin{eqnarray*}
\langle \mathcal {F}^{{\rm approx}}_{{\text{XEB},\text{top-}k}}(\mathcal {S})\rangle = F \ln (|\tilde{\mathcal {S}}|/k),
\end{eqnarray*}

where $F$ is, again, the fidelity of the classical simulation. The factor $F$ in Equation (4) can be heuristically understood as the probability of the post-processed samples according to $p(s)$ belonging to the sequence with the top $k$  $q(s)$. By comparing $\langle \mathcal {F}^{{\rm approx}}_{{\text{XEB},\text{top-}k}}(\mathcal {S})\rangle$ to $\langle \mathcal {F}_{{\rm XEB}}^{{\rm approx}}(\mathcal {S})\rangle$, we observe that the amplification factor provided by the top-$k$ method on the XEB value is approximately $\ln (|\tilde{\mathcal {S}}|/k)$. Thus, to reach a certain XEB value, the top-$k$ method reduces the required simulation fidelity by a factor of $[\ln (|\tilde{\mathcal {S}}|/k)]^{-1}$, which directly translates to a reduction in the computational cost by the same factor.

**Figure 3.**
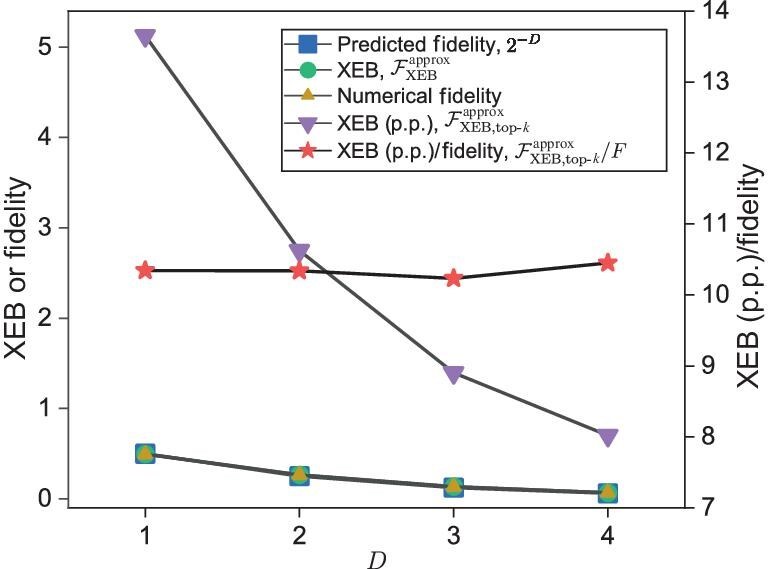
Simulation fidelity (theoretically predicted as well as numerically calculated) and the XEB values of samples obtained via the MCMC sampling method and the top-$k$ method, respectively, under the circumstance of contracting $2^{-D}$ subnetworks for various positive integers $D$. The circuit under consideration consists of 30 qubits with 14 layers of gate operations. The numerical fidelity is computed using approximate amplitudes and exact amplitudes. Here $\mathcal {F}_{\rm XEB}^{\rm approx}$ is the XEB value of the $2^{20}$ samples obtained from $2^{20}\times 2^{6}$ bitstrings via MCMC sampling and $\mathcal {F}_{\text{XEB},\text{top-}k}^{\rm approx}$ is the XEB value of the $2^{15}$ samples selected from $2^{30}$ bitstrings via the top-$k$ method. The post-processing (p.p.) step increases XEB, enabling XEB to exceed 1 for circuits with a small $D$ value.

**Figure 4.**
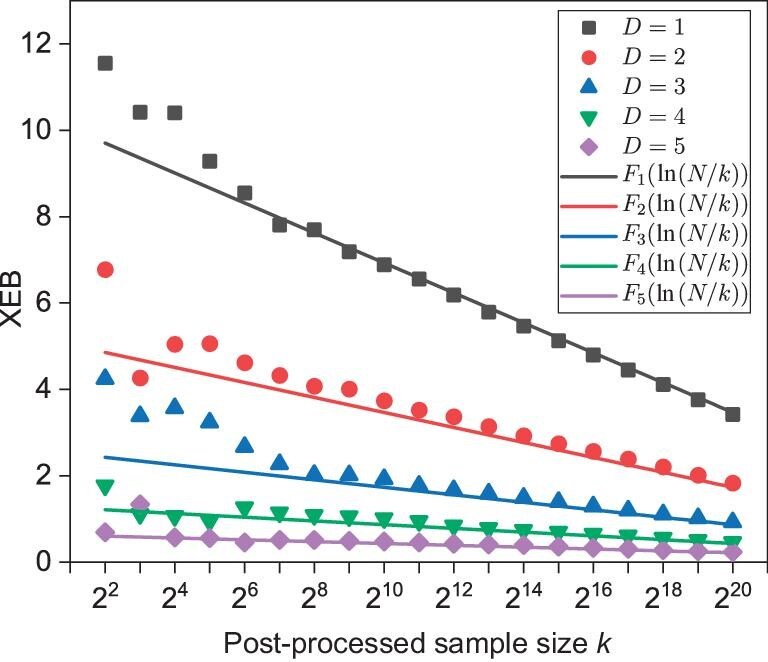
The theoretical and numerical results of XEB (p.p.) are presented for different subnetwork contraction fractions $f=2^{-D}$, where $D$ increases from 1 to 5. The theoretical results are calculated using Equation (4), where $F_D \approx 2^{-D}$ represents the fidelity and $N=|\tilde{\mathcal {S}}|=2^{30}$ is the sequence size.

To validate our expectations, we perform numerical experiments on a small-scale random circuit, structurally similar to the *Sycamore* circuit, on 30 qubits with 14 layers of gates. In all numerical experiments, we use the approximate simulation method described in the previous section to evaluate output probabilities of strings in $\tilde{\mathcal {S}}$. First, for MCMC sampling, we create $\tilde{\mathcal {S}}$ with $|\tilde{\mathcal {S}}|=2^{20}\times 2^{6}$ and obtain $2^{20}$ samples for various degrees of approximation controlled by contracting different fractions of the subnetwork. We observe, in accordance with Equation (3) that, for each fraction $f=2^{-D}$ with $D\in \lbrace 1,2,3,4\rbrace$, the XEB value $\mathcal {F}_{{\rm XEB}}^{{\rm approx}}$, numerical fidelity and analytically predicted fidelity $2^{-D}$ all agree well with each other (Fig. 3). As for sampling via the top-$k$ method, we simply select $k=2^{15}$ strings with the top-$k$ greatest $p(x)$ values from $2^{30}$ strings as the samples. We observe that $\mathcal {F}^{{\rm approx}}_{{\text{XEB},\text{top-}k}}/F$ centers around the same value for all $D$ (Fig. 3) and calculate the numerical value of $\mathcal {F}^{{\rm approx}}_{{\text{XEB},\text{top-}k}}/F$ to be $10.4694 \pm 0.2635$, which is close to the value $\ln 2^{15}=10.3972$ predicted by Equation (4). We also examine the dependence of $\mathcal {F}^{{\rm approx}}_{{\text{XEB},\text{top-}k}}$ on $k$ and observe that, for $k\ge 2^{8}$, the numerical data of $\mathcal {F}^{{\rm approx}}_{{\text{XEB},\text{top-}k}}$ fit extremely well with the lines predicted by Equation (4) for various $D$ (Fig. 4). We expect that the deviation for smaller $k$ from Equation (4) is due to statistical fluctuations more eminent at small sample sizes and that, for larger sample sizes, i.e. larger $k$, Equation (4) should hold well.

**Figure 5.**
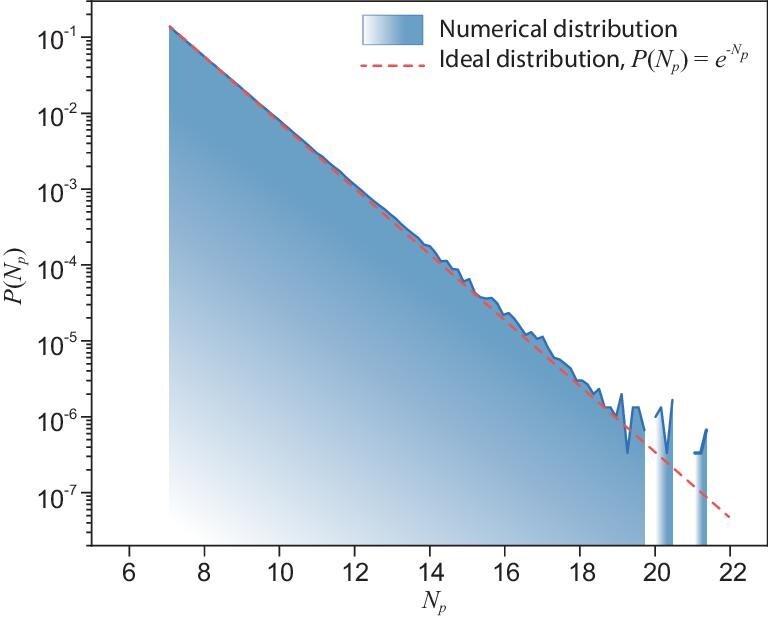
Histogram of the probabilities of three million post-processed samples from the *Sycamore* circuit with 53 qubits and 20 cycles. The probabilities $|\varphi (s)|^2/f$ are normalized according to the fraction of the tensor network to be contracted. The red dotted line denotes the Porter-Thomas distribution.

Figure 5 shows the histogram of normalized circuit-output probabilities $|\varphi (s)|^2/f$, corresponding to the bitstrings in the post-processed samples $\mathcal {S}$, with $\varphi (s)$ the amplitude of bitstring $s$, which is observed to fit the PT distribution well. This is the result of the following two observations. First, the values of the approximate output probabilities $p(s)$ also satisfy the PT distribution [18]. Secondly, the top-$k$ values selected from probabilities with the PT distribution still satisfy the PT distribution.

### Optimizing the tensor contraction

Contraction of complex tensor networks is a difficult task, as different contraction pathways require significantly different computational resources. When simulating quantum advantage experiments using tensor networks, the primary challenge lies in finding the most computationally efficient contraction path within the constraints of limited memory. Pioneering efforts have made substantial progress, such as simplifying tensor networks based on outcome bitstrings and optimizing contraction paths through simulated annealing searches.

The slicing technique [28–31] allows for the reduction of memory consumption during contractions to fit within available device memory. Tensor network slicing involves selectively calculating only one of the two possible values for certain edges within the network during contractions, and then summing all possible value combinations at the end. For example, when removing edges $i$ and $j$ from tensor network TN, we have


\begin{eqnarray*}
{\rm TN} &=& {\rm TN}_{i=0, j=0} + {\rm TN}_{i=0, j=1}\\
&&+ {\rm TN}_{i=1, j=0} + {\rm TN}_{i=1,\, j=1} .
\end{eqnarray*}

**Figure 6.**
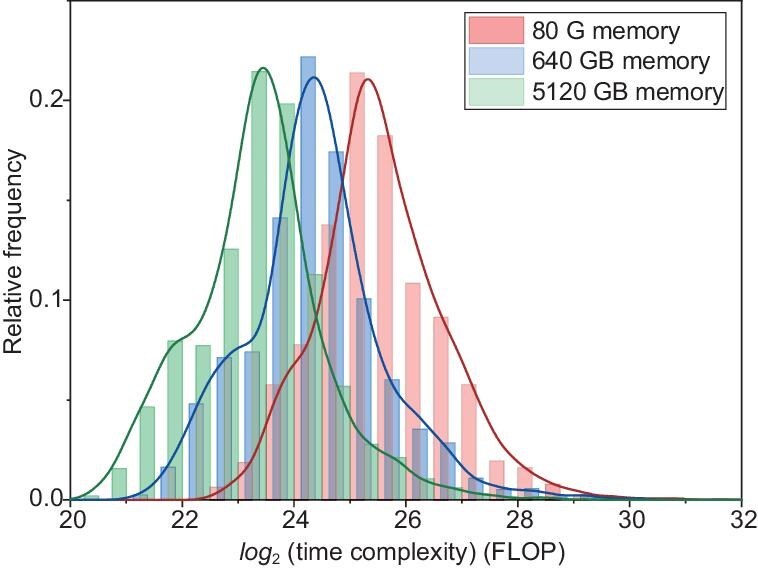
The time complexity distribution of contraction paths is examined under memory constraints of 80, 640 and 5120 GB. Notably, as the available memory increases by a factor of 8, the time complexity of the optimal contraction path decreases by half. This observation highlights the impact of memory resources on the efficiency of contraction operations.

Slicing reduces the number of indices during tensor contraction, lowering memory consumption. However, sliced edges are the last to be contracted, restricting the flexibility of contraction pathways and resulting in increased computational resources. This is essentially a trade-off between time and space. Numerical experiments show that, under memory constraints of 80, 640 and 5120 GB, the computational time complexity of the optimal contraction path is approximately inversely proportional to the maximum memory size, shown in Fig. 6. Tensor contractions are fetch-intensive tasks. We use 80-GB A100 GPUs with an intra-GPU memory bandwidth of 2 TB/s. Eight A100 GPUs within each node are interconnected via NVLink with an intra-GPU speed of 600 GB/s, while nodes are connected via InfiniBand with an intra-node speed of 25 GB/s. We adopt a node-level computation approach, using $8\times 80 = 640\, \text{GB}$ of memory for contraction computations to achieve a balance between calculation and communication.

We visualize the optimal contraction tree, which consists of a stem path leading from a leaf node to the root node. Nodes on the stem path consume significantly more computation and memory resources than other nodes. To reduce inter-GPU communication, we distributed tensors of the stem nodes across eight GPUs based on the first three dimensions. During a single tensor contraction, if the first three dimensions are not contracted, no inter-GPU communication is required, and each of the eight GPUs computes its respective part. If some of the first three dimensions are contracted, we permute the tensor, swapping the first three dimensions with the next three, and distribute the tensor accordingly with the new three dimensions. To minimize communication overhead, we optimize the order of dimensions within the stem path, ensuring that the three dimensions used for distributed storage persist through as many steps as possible without undergoing contraction. In the final stages of the stem path, we follow the method by Pan *et al.* [18], calculating only the parts contributing to the final bitstrings.

We observe that the order of dimensions significantly affects the computation time of cuTensor. Empirically, better performance is achieved when satisfying the following conditions:

1. placing the contracted dimensions of input tensors at the end,
2. ensuring the same order of the contracted dimensions between two input tensors,
3. arranging the order of dimensions of input tensors according to the order of dimensions of output tensors.

We employ a greedy algorithm to optimize the order of dimensions of all tensors within the contraction tree, starting from the root node. Additionally, we use cuTensor’s einsum [26] in the contraction process. For each contraction step, we compared the performance of transpose-transpose-GEMM-transpose and cuTensor and chose to use the faster of the two.

Our algorithm utilizes eight GPUs on a single node for computation, with each node handling and accumulating different sliced subtasks. To obtain the final result, we adopt the NVIDIA Collective Communication Library reduction based on the ring-reduce method. Its communication complexity is $2(N-1)\times K/N$, where $K$ represents the data volume per node and $N$ is the number of nodes. Therefore, as the number of nodes increases, the communication time tends toward a constant value, which in our tests is approximately 2 s. Hence, reduction time does not become a main bottleneck.

For the case of an 80-GB memory constraint with a single A100 GPU, we find that the optimal contraction scheme comprises $2^{30}$ subtasks, with each subtask taking 3.95 s to compute. With the constraint of 640-GB memory and eight A100 GPUs, the optimal scheme consists of $2^{24}$ subtasks, and each subtask takes 2.95 s to compute on a single node (eight A100 GPUs). Therefore, we estimate that our optimized eight-GPU parallel algorithm yields a speedup of $3.95 \times 2^6 / (2.95 \times 8) = 10.7$ times.

### Parallelism and complexity for simulating the *Sycamore* circuit

**Figure 7.**
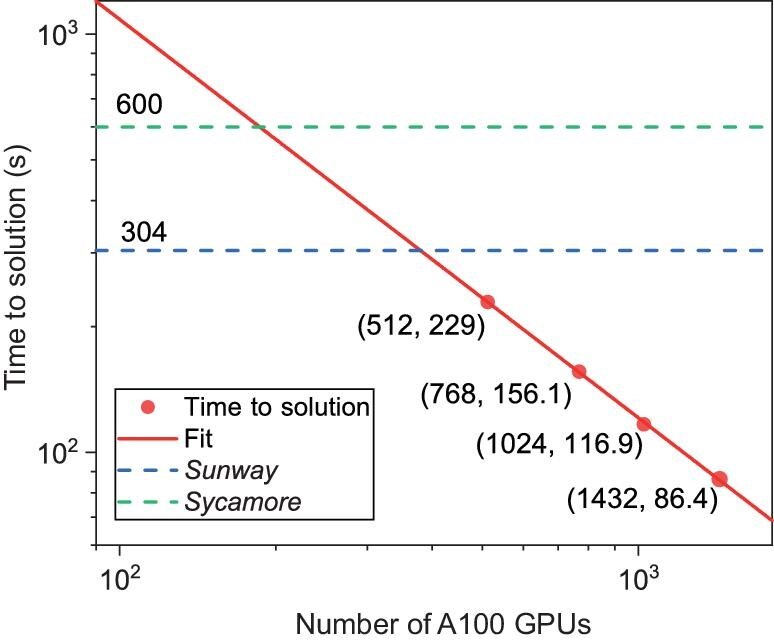
The time to solution for simulating a quantum RCS experiment with ${\rm XEB }= 0.002$ for samples obtained via the post-processing method on/using different numbers of GPUs. The time to solution decreases linearly as the number of GPUs increases, which demonstrates the parallelism of our algorithm.

To sample from the *Sycamore* circuit, we choose 10 qubits as open qubits to generate $\tilde{\mathcal {S}}$ with $|\tilde{\mathcal {S}}|=(3\times 10^{6})\times 2^{10}$ and aim to obtain $k=3\times 10^{6}$ uncorrelated samples via the top-$k$ method. As mentioned above, the large tensor network could be split into independent subnetworks and each subnetwork can be contracted on a single GPU. In our implementation, thanks to the approximate simulation and the post-processing method, we only need to perform $0.03\%$ of the total tasks (i.e. contract $0.03\%$ subnetworks of the total networks) out of a total of $2^{24}$ subtasks to reach an XEB value of 0.002, where each contraction subtask requires $1.1029\times 10^{14}$ FLOPS and can be completed in 3.09 s on eight NVIDIA A100 GPUs. Furthermore, we observe a linear decay of the total time to solution as we increase the number of GPUs used in the computation (Fig. 7), which clearly demonstrates the parallelism of the implementation of our algorithm. In particular, when using 1432 GPUs, the total time to solution for sampling $3\times 10^6$ bitstrings is 86.4 s, which is less than *Sycamore*’s 600-s time to solution.

To verify the fidelity, we utilize the exact output amplitudes from a previous study [33] to calculate the fidelity of our simulation of the *Sycamore* circuit with 53 qubits and 20 layers of gates, and we observe that the numerical fidelity $F_{\text{num}} = 0.018\, 23\%$ fits well with our prediction $F_{\text{predicted}}=0.019\, 07\%$ according to Equation (4).

The complexity of this task is summarized in Table 2. The space complexity of our algorithm is proportional to $s2^M$, where $s$ represents the size of the data type and $M$ corresponds to the targeted contraction treewidth, which can be specified manually in our slicing algorithm. So, the space complexity is completely under control.

**Table 2.** The complexity of simulating the quantum RCS experiment. The task with ${\rm XEB}_{\rm p.p.}=0.002$ corresponds to the simulation of *Sycamore* [9]. The efficiency is calculated as $8 T_c/(P t)$, where 8 is the complex number product factor, $T_c$ is the time complexity, $P$ is the single-precision performance of GPU and $t$ is the time to solution.

	Each subnetwork	*Sycamore* (${\rm XEB}_{\rm p.p.}=0.002$)	${\rm Fidelity} =1$
Time complexity (FLOP)	$ 1.1029 \times 10^{14}$	$ 5.5527\times 10^{17}$	$ 1.8504\times 10^{21}$
Space complexity (GB)	640	640	640
Memory complexity (bytes)	$ 1.5200\times 10^{12}$	$ 7.6182\times 10^{15}$	$ 2.5668\times 10^{19}$
Efficiency (%)	23.57	22.12	–
Time to solution (s)	2.95	86.4	–
Computer resource (A100)	8	1,432	–

## CONCLUSIONS

We amplified the XEB nearly 7$\times$ using the post-selection algorithm because the post-selection algorithm can filter out high-XEB samples from low-fidelity pre-samples, while low-fidelity sampling only has low complexity. In addition, according to the observation that the computational complexity of the optimal contraction scheme is inversely proportional to the storage space, 8-GPU interconnection technology is developed to achieve 640-GB memory space. We took advantage of the large storage space to increase the speed of parallel algorithms by 10.7$\times$. To complete the same task as *Sycamore*, we ran our algorithm on 1432 NVIDIA A100 GPUs and obtained three million uncorrelated samples with XEB values of $2\times 10^{-3}$ in 86.4 s with an energy consumption of 13.7 kWh. In this sense, we estimate that approximately 206 GPUs have an equivalent computational power in implementing a 53-qubit 20-depth random circuit sampling as *Sycamore* (Fig. 7). With further optimizations, the power consumption is expected to be reduced below that of *Sycamore*. Our results suggest that establishing quantum advantage requires larger-scale quantum experiments.

## Data Availability

We utilized the quantum platform MindSpore Quantum [34] in our experiments. The data and codes that support the findings of this study are openly available in Leapfroggine-Sycamore-53-qubit-RCS and Mindquantum.
